# Effects of Ritonavir, Lopinavir, and Alcohol on ABC Transporters and Secretion of Bile Acid and Bilirubin in Senescent Hepatocytes

**DOI:** 10.3390/ijms27031189

**Published:** 2026-01-25

**Authors:** Liting Chen, Eric Duran, Diego Headrick, Cheng Ji

**Affiliations:** Keck School of Medicine, University of Southern California, Los Angeles, CA 90089, USA

**Keywords:** alcohol/drug use disorders, anti-HIV drugs, hepatocyte bile acid secretion, bilirubin secretion, ABC transporters

## Abstract

Drug- and alcohol-induced liver injury involves impaired bile acids or bilirubin secretion, but it is not known how senescence influences the secretion of hepatocytes exposed to drugs and alcohol. In this study, the toxic effects of ritonavir, lopinavir, and alcohol on hepatocyte transporters and the secretion of bile acids and bilirubin were investigated in hydrogen peroxide-induced senescent HepG2 and doxorubicin-induced senescent primary human hepatocytes. In HepG2, intracellular conjugated bilirubin increased upon senescence and extracellular conjugated bilirubin in culture medium was decreased by ritonavir and lopinavir treatment. In the primary hepatocytes, intracellular bile acids or medium bilirubin were not significantly changed upon senescence. However, intracellular bile acids were increased, and medium conjugated bilirubin were decreased in senescent primary hepatocytes treated with alcohol and the two drugs. Transcriptional expressions of adenosine triphosphate (ATP)-binding cassette (ABC) transporters (ABCB4, ABCC6, ABCB11, and ABCD3) were decreased whereas UDP-glucuronosyltransferase (UGT1A1) was increased by ritonavir and lopinavir in senescent HepG2. In senescent primary hepatocytes, expressions of ABCB11, ABCC1, ABCC2, ABCC3, ABCC4, and ABCC6 were apparently reduced whereas UGT1A1 and the cytochrome P450 enzyme CYP7A1 were markedly increased by alcohol combined with ritonavir and lopinavir. Selective ABCC6 knockdown in the primary hepatocytes altered expressions of two senescence markers, Lamin A/C and cyclin-dependent kinase inhibitor CKI (p21), increased expressions of CYP7A1 and hydroxy methyl glutaryl-CoA reductase (HMGCR), and increased intracellular bile acids. Further, anti-cholestasis agents, ursodeoxycholic acid and glycyrrhizin, significantly ameliorated the impaired secretions of bile acids and bilirubin as well as reducing intracellular lipid accumulation and cell death caused by ritonavir, lopinavir, and alcohol in the primary hepatocytes with ABCC6 knockdown. These results indicate that senescence moderately impairs the ABC transporters of hepatocytes and secretion of bile acids or bilirubin, which become worse in the presence of the drugs and alcohol but could be improved by anti-cholestasis agents.

## 1. Introduction

The population aged 60 and older worldwide is increasing, which gives rise to significant new healthcare challenges such as the excessive use of prescription medications and increased rates of alcohol consumption or combined drug–alcohol exposure among the elderly due to social isolation or depression [1]. Aging-related decline in major human organs including the liver is rising. A leading cause of liver injury is alcohol and/or drug uses that contribute to rising rates of morbidity and mortality [2,3]. Alcohol-/drug-induced hepatotoxicity involves cellular oxidative stress, mitochondrial stress, endoplasmic reticulum stress, and inflammation, which could be hepatocellular, cholestatic, or mixed. One of the important mechanisms underlying the drug-/alcohol-induced hepatotoxicity is the impaired secretion of bile acids and bilirubin that leads to the toxic accumulation of bile acids and/or bilirubin within hepatocytes and subsequent liver cell death [3,4]. The secretion of bile acids and bilirubin involves basolateral and canicular transporters such as the adenosine triphosphate (ATP)-binding cassette (ABC) transporters, key factors in cholesterol and bile acid synthesis and metabolism such as the hydroxy methyl glutaryl-CoA reductase (HMGCR) and cytochrome P450 CPY7A1, bilirubin conjugation enzymes such as the enzyme glucuronyltransferase (UGT1A1), and the nuclear farnesoid X receptor (FXR). Previous studies from us and others indicate that drugs/alcohol disrupt bile acid uptake, process, and transport, contributing to cholestatic disease in animal models [5,6,7]. In mice intoxicated with ethanol, the elevation of the total bile acid pool including free taurine- and glycine-conjugated bile acids was observed [6,7]. Expressions of CYP7A1 along with bile acid excretion transporters including ABCB1, ABCB11, and ABCC2 were upregulated whereas the liver bile acid uptake transporter LBAT/NTCB and nuclear receptor FXR were downregulated by alcohol [7,8]. The anti-HIV protease inhibitors, ritonavir and lopinavir, used in current regimens are potent inhibitors of cytochrome P450 CYP3A4 and CYP2D6, which consequently affects the metabolism of bile acids and bilirubin [4]. Ritonavir and lopinavir also inhibit bile acid transporters, including ABCB3, ABCB11/BSEP (bile salt export pump), ABCC2, and the organic anion transporting polypeptides OATP1B1 and OATP1B3, which disturb the secretion and homeostasis of bile acids and bilirubin [4]. In senescent hepatocytes or aging liver, evidence has emerged that there are adverse effects of drugs and alcohol on the ABC transporters and bile acid/bilirubin secretion. A recent clinical case report indicated that, in an elderly patient, ritonavir and lopinavir were associated with persistent hepatic secretion failure, which might involve the transporter ABCC6 [9]. The ABCC6 defect has been identified as a cause for an autosomal recessive disease termed pseudoxanthoma elasticum (PXE) [10], which is also associated with disorders of cholesterol/bile acid metabolism and obstructive jaundice in a PXE-affected 61-year-old female [11]. In addition, aging is reported to disrupt bile acid homeostasis leading to cholestasis, fatty liver diseases, and hepatic carcinogenesis [12]. These lines of evidence prompt us to hypothesize that aging is an influential factor for the drug-/alcohol-induced secretion impairment of bile acids and bilirubin and consequent hepatic injury. Senescence could be induced by different stresses and oxidative stress is the most common stress factor [13,14]. The pro-oxidant hydrogen peroxide (H_2_O_2_) has been used to induce cell senescence in in vitro models and doxorubicin is also a pro-oxidant, which at lower doses has recently been used for senescence induction of more physiologically relevant primary human cells as well as for liver aging induction in animal models, which is associated with the development of metabolic dysfunction-associated steatotic liver disease (MASLD) [15,16,17]. In this study, we applied either H_2_O_2_ or doxorubicin as the senescence inducer and found evidence supporting that in senescent human hepatocytes, ritonavir, lopinavir, and alcohol have detrimental impacts on ABC transporters, bile acid/bilirubin metabolic enzymes, and the secretion of bile acids and bilirubin.

## 2. Results

### 2.1. Effects of Ritonavir, Lopinavir, and Alcohol on Secretion of Bile Acids or Bilirubin in Senescent Hepatocytes

**Figure 1:**
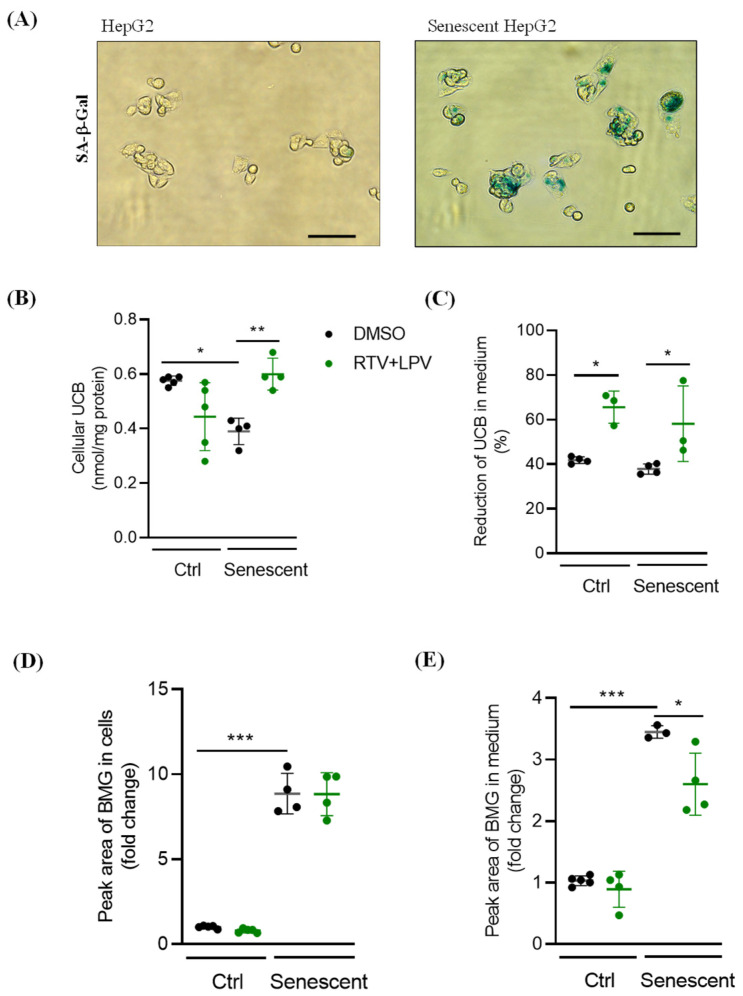
Effects of ritonavir and lopinavir on bile acid and bilirubin secretion in senescent HepG2 cells. (**A**) HepG2 cells stained with senescence-associated β-galactosidase (SA-β-gal) (green color); (**B**) cellular unconjugated bilirubin (UCB); (**C**) reduction in UCB in the culture medium compared to non-senescent control; (**D**) cellular conjugated bilirubin monoglucuronide (BMG); (**E**) BMG in culture medium; Ctrl, non-senescent HepG2; Senescent, senescent HepG2; DMSO, dimethylsulfoxide as vehicle control; RTV, ritonavir; LPV, lopinavir; RTV + LPV, ritonavir plus lopinavir; Scale bar, 90 mM; *, *p* < 0.05; **, *p* < 0.01; ***, *p* < 0.005.

One of the important biological functions of hepatocytes is the secretion of bile acids and conjugated bilirubin, which contributes to bile acid/bilirubin homeostasis. Either ritonavir (RTV) or lopinavir (LPV) has been shown to interfere with the secretion of bile acids or conjugated bilirubin leading to the accumulation of bile acids inside the liver cells or elevation of serum bilirubin [2,4,5]. Senescent hepatocytes were induced to test how the effects of ritonavir and lopinavir on the secretion were different. Senescence of HepG2 was induced by treating the cells with hydrogen peroxide, which was indicated by an increased expression of senescence-associated β-galactosidase (SA-β-gal) (Figure 1A). The HepG2 cells were treated with RTV and LPV and then incubated in fresh media containing bilirubin for five hours before secretion measurements. Levels of cellular unconjugated bilirubin (UCB) were decreased in senescent HepG2 compared to the non-senescent control (Figure 1B). RTV and LPV treatments increased the cellular UCB in the senescent cells but not in the control cells. There was a significant difference in UCB between the drug-treated control and senescent HepG2. In the culture media, the UCB levels were not changed between control and senescent cells but, upon treatment with the anti-HIV drugs, UCB levels in the medium were reduced in control or senescent cells and there was no difference between control and senescence (Figure 1C). Conjugated bilirubin monoglucuronide (BMG) increased dramatically inside the senescent HepG2 compared to control HepG2, and RTV and LPV did not affect further the BMG levels in either control or senescent cells (Figure 1D). The BMG levels were markedly increased in the media upon senescence, which were decreased by RTV and LPV in the senescent HepG2 (Figure 1E).

**Figure 2:**
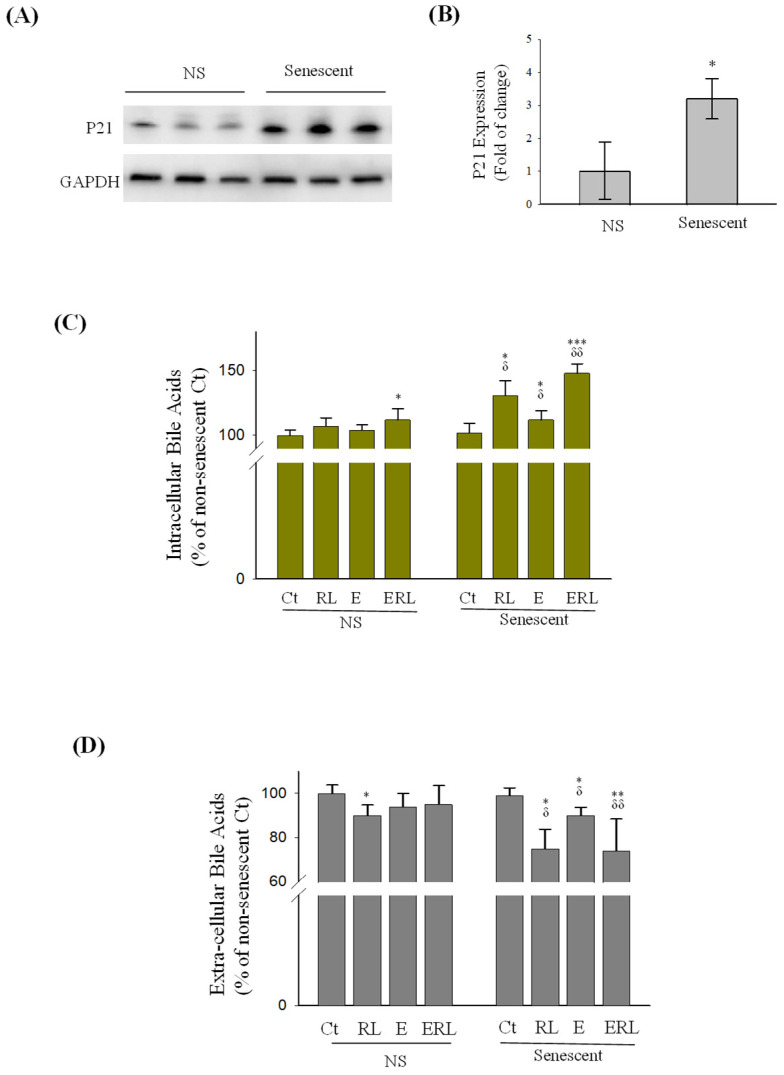
Effects of anti-HIV drugs and alcohol on bile acid secretion in senescent primary human hepatocytes. (**A**) Western blots of cyclin-dependent kinase inhibitor CKI (p21). GAPDH, glyceraldehyde 3-phosphate dehydrogenase as an internal control; lanes 1, 2, and 3 were loaded with protein samples from triplicate non-senescent (NS) primary human hepatocytes (PHHs), and lanes 4, 5, and 6 were loaded with protein samples from triplicate senescent PHHs (Senescent). (**B**) RT-PCR of p21 mRNA, *, *p* < 0.05; (**C**) intracellular bile acids; (**D**) bile acids in cell culture medium; Ct, vehicle control; RL, ritonavir plus lopinavir; E, ethanol; ERL, ritonavir, lopinavir and ethanol; *, *p* < 0.05; **, *p* < 0.01; ***, *p* < 0.005 compared to Ct of the same cell group; ^δ^, *p* < 0.05 and ^δδ^, *p* < 0.01 compared to the same treatment between non-senescent and senescent cells.

Premature senescence was induced in primary human hepatocytes (PHHs) by treating cells with doxorubicin, which had increased the expression of cyclin-dependent kinase inhibitor CKI (p21) (Figure 2A,B). Before measuring the secretions, the PHHs were treated with RTV, LPV, and/or ethanol and then incubated in fresh media containing cholic acid for five hours. The status of senescence of PHHs had little effect on the levels of intracellular bile acids (Figure 2C). In the non-senescent PHHs, the levels of intracellular bile acids were increased significantly by ethanol combined with RTV or LPV but were not affected by RTV, LPV, or ethanol alone. In the senescent PHHs, the levels of intracellular bile acids were increased significantly by RTV and LPV or by ethanol alone. Alcohol and the drugs together had synergistic effects on the intracellular bile acids of senescent PHHs, which were increased by more than 50%. The extracellular bile acids in the medium of non-senescent PHHs were decreased slightly by RTV and LPV (Figure 2D), whereas in the senescent PHHs, the extracellular bile acids were decreased by RTV and LPV, or ethanol alone, which were decreased further by the drug–ethanol combination, indicating a severe secretion impairment of bile acids in the senescent primary hepatocytes.

### 2.2. Differential Expressions of Transporters in Senescent PHHs Treated with RTV, LPV, and/or Alcohol

**Figure 3:**
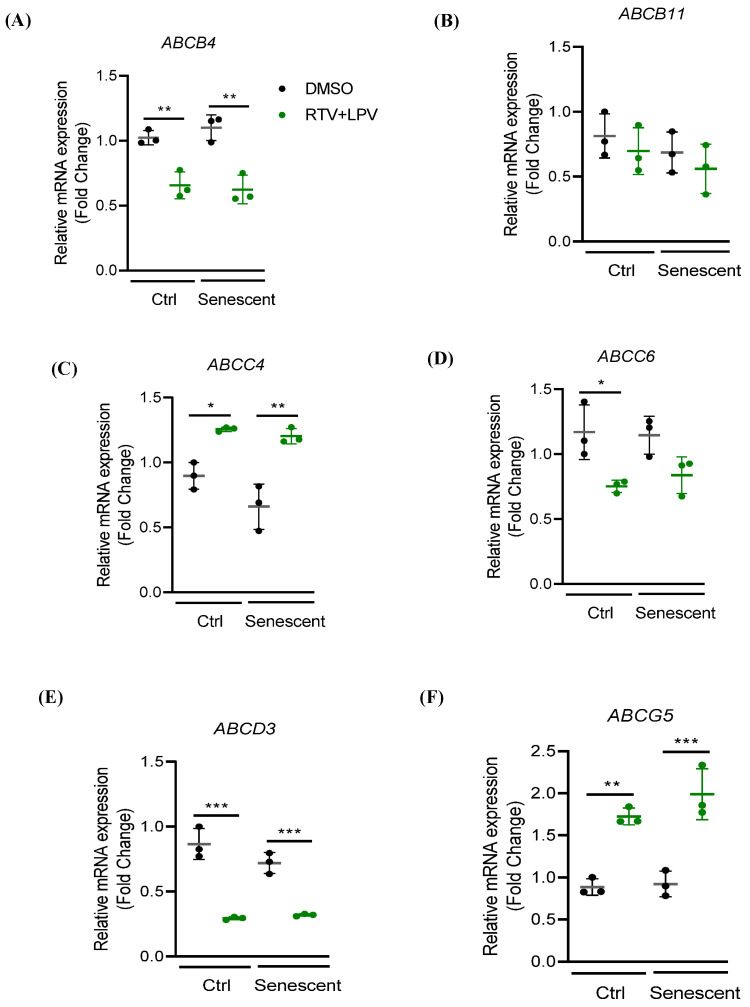
Expressions of selective ABC transporters in HepG2 treated with anti-HIV drugs. Ctrl, non-senescent control HepG2; Senescent, senescent HepG2; DMSO, dimethyl sulfoxide as vehicle control; RTV, ritonavir; LPV, lopinavir; RTV + LPV, ritonavir plus lopinavir; *, *p* < 0.05; **, *p* < 0.01; ***, *p* < 0.005.

**Figure 4:**
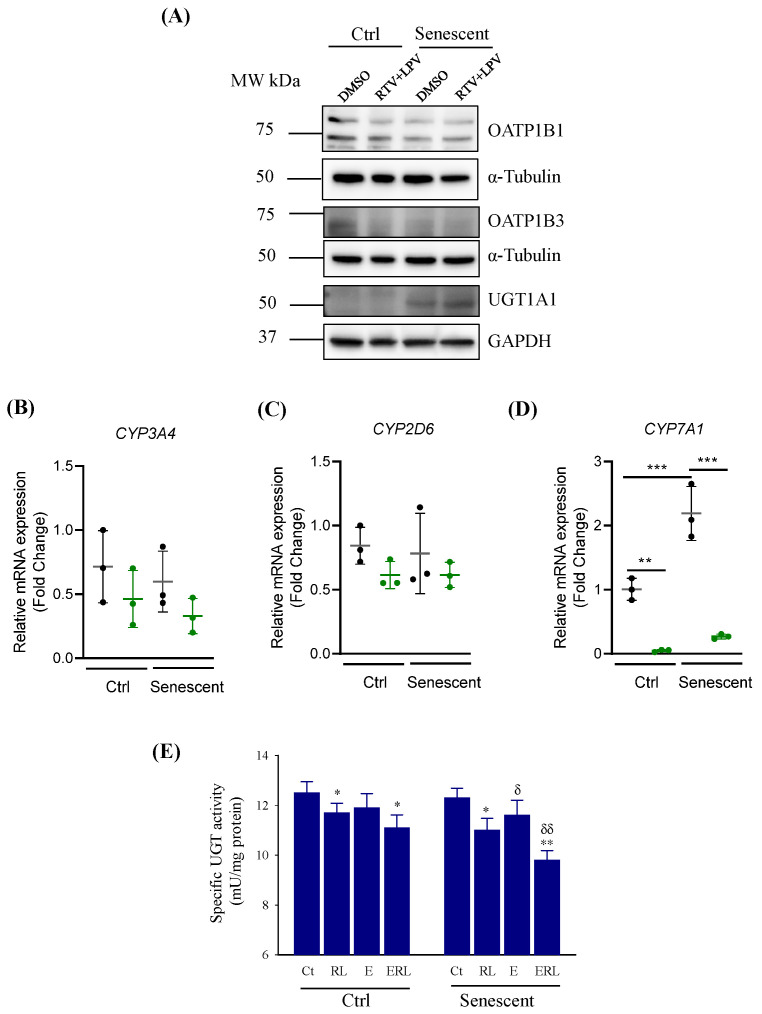
Effects of anti-HIV drugs on expression of selective factors related to hepatic metabolism or secretion of bile acid or bilirubin. (**A**) Western blots of proteins from HepG2; Ctrl, non-senescent HepG2; Senescent, senescent HepG2; DMSO, dimethyl sulfoxide as vehicle control; RTV, ritonavir; LPV, lopinavir; RTV + LPV, ritonavir plus lopinavir; OPTP1B1 and OATPIB3, organic anion transporter polypeptide member 1B1 and 1B3; GAPDH, glyceraldehyde 3-phosphate dehydrogenase as an internal control; UGT1A1, UDP-glucuronosyltransferase (UGT) 1A1; (**B**–**D**), RT-PCR of mRNAs from HepG2; CYP3A4, CYP2D6, and CYP7A1: cytochrome P450 3A4, 2D6, and 7A1, respectively; Green dots indicate drug treatments; **, *p* < 0.01; ***, *p* < 0.005; (**E**) Enzyme activities of UGT from non-senescent PHHs (Ctrl) or senescent PHHs (Senescent); *, *p* < 0.05; **, *p* < 0.01 compared to Ct of the same cell group; ^δ^, *p* < 0.05 and ^δδ^, *p* < 0.01 compared to the same treatment between non-senescent and senescent PHHs.

Factors that might potentially cause the intracellular accumulation and secretion impairment of bile acids or bilirubin include the adenosine triphosphate (ATP)-binding cassette (ABC) transporters (ABCB1, ABCB4, ABCB11, ABCC1, ABCC2, ABCC3, ABCC4, ABCC6, ABCD3, and ABCG5), the hepatic organic anion transporting polypeptide members (OATP1B1 and OATP1B3), the cytochrome P450 enzymes (CYP3A4, CYP7A1, and CYP2D6), and the UDP-glucuronosyltransferase UGT1A1 [3,4,5,18,19]. Based on RT-PCR analysis, mRNA expressions of *ABCB1* (Figure S1A), *ABCB4* (Figure 3A), or *ABCB11* (Figure 3B) were not changed significantly upon senescence of HepG2. RTV and LPV treatments increased *ABCB1* only in senescent HepG2, inhibited *ABCB4* in both control and senescent cells, and inhibited *ABCB11* slightly in control and senescent cells. Expressions of *ABCC1* (Figure S1B), *ABCC2* (Figure S1C), *ABCC3* (Figure S1D), *ABCC4* (Figure 3C), and *ABCC6* (Figure 3D) were not affected by senescence alone without drug challenge. RTV and LPV treatments increased the expressions of *ABCC2*, *ABCC3*, and *ABCC4* in both control and senescent cells, and the expressions of *ABCC2*, *ABCC3*, and *ABCC4* were increased more in senescent cells compared to control cells. The expression of *ABCC6* was inhibited in either control or senescent HepG2 treated with RTV and LPV. The expression of *ABCD3* was not affected by senescence but was inhibited by the RTV and LPV treatment (Figure 3E). The expression of *ABCG5* was increased in both control and senescent HepG2, and the increased *ABCG5* was more in senescent cells than in control cells (Figure 3F). No significant changes for OATP1B1 or OATP1B3 were detected with immunoblotting analysis between control and senescent HepG2 or between RTV and LPV treatments. However, UGT1A1 was apparently increased upon senescence based on the immunoblotting analysis (Figure 4A). Neither senescence nor RTV and LPV treatments significantly affected the expression of *CYP3A4* or *CYP2D6* in HepG2 (Figure 4B,C). The mRNA expression of *CYP7A1* was increased upon senescence but inhibited markedly by the anti-HIV drugs (Figure 4D).

In the primary human hepatocytes, the enzyme activities of UGT1A1 were inhibited by 6.2% by RTV–LPV and by 4.5% by alcohol alone, which were reduced by 11.5% in the presence of alcohol combined with RTV and LPV (Figure 4E). There was a trend of reduction in UGT1A1 activities in senescent PHHs compared to non-senescent PHHs. UGT1A1 activities were inhibited by 10% by RTV–LPV and by 5.7% by alcohol alone, which were reduced by more than 20% in the presence of alcohol combined with RTV and LPV.

**Figure 5:**
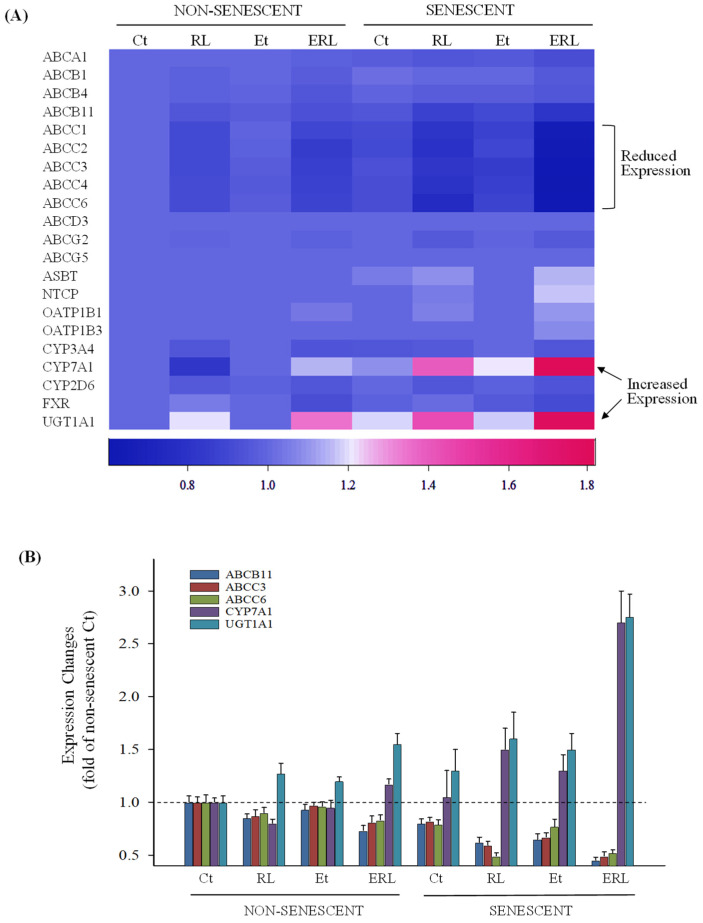
Changes in genes/factors related to metabolism and secretions of bile acids and bilirubin in senescent or non-senescent primary human hepatocytes treated with ritonavir, lopinavir, and alcohol. (**A**) Heat-map of gene expression; (**B**) RT-PCR of selective genes; Ct, dimethyl sulfoxide as vehicle control; RL, ritonavir plus lopinavir; Et, ethanol; ERL, ritonavir, lopinavir, and alcohol.

Gene expressions in PHHs were further evaluated with a large-scale mRNA microarray analysis. The expressions of genes relevant to hepatic transporters, secretions, and drug metabolism/detoxification were clustered in a heat-map for comparisons (Figure 5A). Expressions of *ABCA1*, *ABCB1*, *ABCB4*, *ABCD3*, *ABCG2*, and *ABCG5* were not changed significantly between non-senescent and senescent PHHs or between alcohol and drug treatments. Expressions of *ABCB11*, *ABCC1*, *ABCC2*, *ABCC3*, *ABCC4*, and *ABCC6* were inhibited by the HIV drugs but not by alcohol alone, which were further inhibited upon senescence or by the alcohol and drug combination treatment. Expressions of *ASBT* (the apical sodium-dependent bile acid transporter), *NTCP* (the basolateral sodium/bile acid cotransporter), *OATP1B1*, and *OATP1B3* in the senescent PHHs were slightly increased by RTV–LPV and moderately increased by the combination treatment with alcohol and the drugs. No consistent mRNA changes for *CYP3A4*, *CYP2D6*, or farnesoid X receptor (*FXR*), which regulates bile acid homeostasis, were detected between treatments or between senescence and non-senescence. However, expressions of *CYP7A1* and *UGT1A1* were increased in the control PHHs treated with alcohol combined with the drugs, which were further increased in senescent PHHs. RT-PCR for selected genes was further conducted to confirm the microarray results, and the mRNA changes for *ABCB11*, *ABCC3*, *ABCC6*, *CYP7A1*, and *UGT1A1* could mostly be confirmed by the RT-PCR (Figure 5B).

### 2.3. Effects of RTV, LPV, and Alcohol on Bile Acid and Bilirubin Secretion in PHHs Deficient in ABCC6

**Figure 6:**
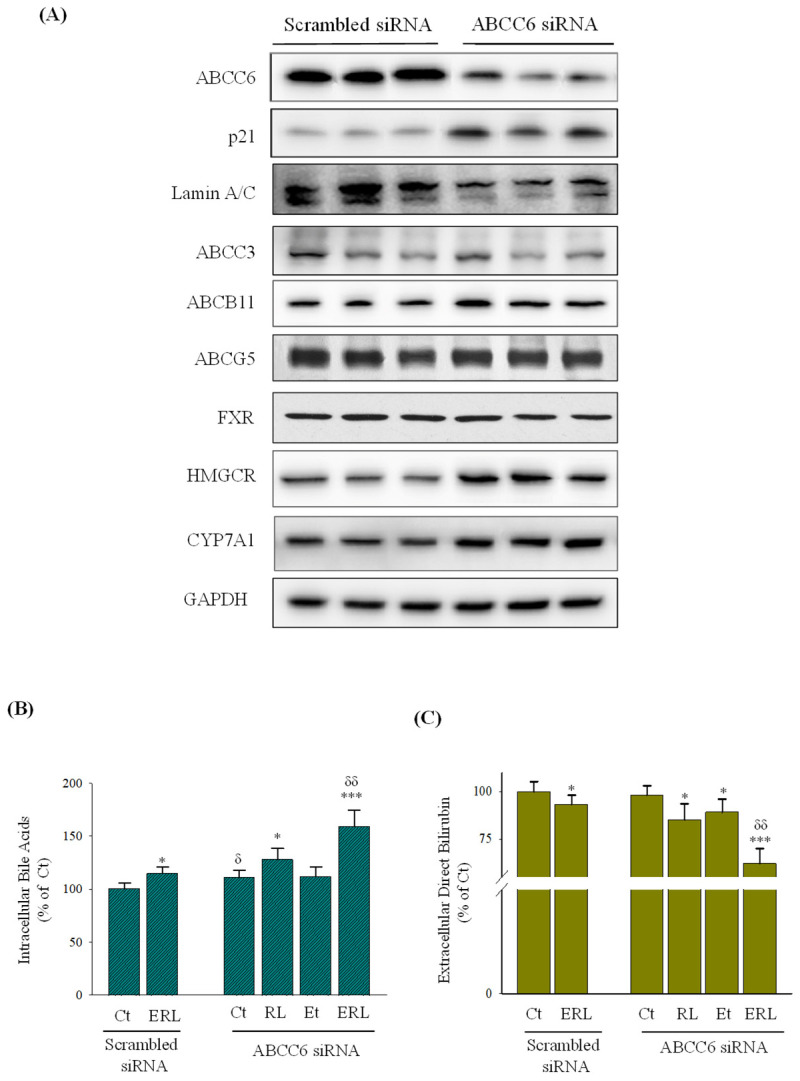
Effects of ABCC6 knockdown on secretions of bile acids and bilirubin in primary human hepatocytes treated with ritonavir, lopinavir, and alcohol. (**A**) Immunoblots of proteins from primary human hepatocytes (PHHs) with ABCC6 being knocked down by small RNA (siRNA) interference; lanes 1, 2, and 3 were loaded with protein samples from triplicate primary human hepatocytes treated with control scrambled siRNA, and lanes 4, 5, and 6 were loaded with protein samples from triplicate primary human hepatocytes treated with ABCC6 siRNA. HMGCR, 3-hydroxy-3-methylglutaryl-CoA reductase. (**B**) Relative intracellular levels of bile acids; (**C**) relative levels of conjugated bilirubin in the culture medium; Ct, PHHs with scramble siRNA or ABCC6 siRNA and treated with dimethyl sulfoxide; RL, PHHs treated with ritonavir plus lopinavir; Et, PHHs treated with ethanol; ERL, PHHs treated with ritonavir, lopinavir, and alcohol; *, *p* < 0.05; *** and *p* < 0.005 compared to Ct of the same cell group; ^δ^, *p* < 0.05 and ^δδ^, *p* < 0.01 compared to the same treatment between PHHs with and without ABCC6 being knocked down.

The C-class of the ABC transporters was suppressed most by the drug and alcohol treatments in the senescent hepatocytes. To confirm further the effects of senescence on the drug- and alcohol-induced secretion impairments, we examined the potential consequences of ABCC6 knockdown in primary human hepatocytes considering that senescence could be induced in HepG2 deficient in ABCC6 [20] and that an indirect role of ABCC6 in RTV-/LPV-induced severe persistent hepatocellular secretory failure (PHSF) was reported in a recent clinical case report [9,11]. To that end, *ABCC6* was knocked down in PHHs with small interfering RNA (siRNA) (Figure 6A). The expression of p21 protein was upregulated and the expression of Lamin A/C was downregulated, indicating that the PHHs with the ABCC6 knockdown were at a status of senescence. The ABCC6 knockdown did not affect the expression of the same class transporter, ABCC3, but exerted mixed effects on expressions of other class transporters, ABCB11 and ABCG5. ABCB11 was slightly increased by the knockdown whereas ABCG5 was not changed. In addition, upon ABCC6 knockdown, hydroxy methyl glutaryl-CoA reductase (HMGCR) and CYP7A1 which are necessary for the hepatic biosynthesis of cholesterol and bile acids were moderately increased, whereas nuclear receptor FXR was not changed (Figure 6A).

The ABCC6 knockdown had an impact on the secretion of bile acids and bilirubin. Intracellular bile acids (i.e., bile acid accumulation) were increased in PHHs with ABCC6 knockdown compared to PHHs without ABCC6 knockdown, which was further increased by 16% by the RTV and LPV treatment (Figure 6B). Compared to the RTV and LPV treatment, ethanol treatment did not increase the levels of intracellular bile acids significantly in the PHHs with ABCC6 knockdown. However, alcohol combined with RTV and LPV increased the bile acid levels by nearly 50% PHHs with ABCC6 knockdown compared to PHHs without ABCC6 knockdown. Extracellular direct bilirubin (i.e., conjugated bilirubin) was not significantly changed between PHHs with and without ABCC6 knockdown or any drugs or alcohol treatments (Figure 6C). The extracellular levels of direct bilirubin were reduced by 15% when the ABCC6-deficient PHHs were treated with RTV and LPV, by 11% when treated with ethanol, and by 48% when treated with ethanol combined with the drugs. There was a synergistic effect of alcohol and drugs on the bilirubin secretion in the PHHs deficient in ABCC6, supporting the role of ABCC6 deficiency or senescence in the drug- and alcohol-induced secretion impairment.

### 2.4. Effects of Anti-Cholestasis Drugs on Bile Acid and Bilirubin Secretions in Senescent PHHs

**Figure 7:**
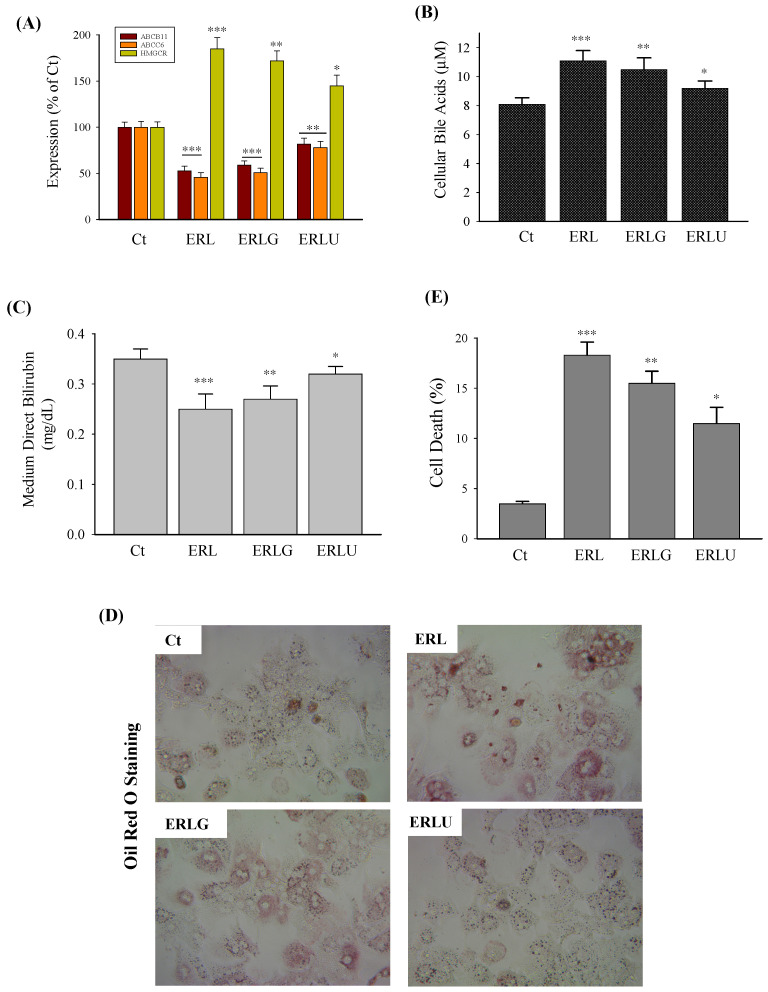
Protective effects of anti-cholestasis drugs on bile acid/bilirubin secretion and cell injury in senescent primary human hepatocytes treated with ritonavir, lopinavir, and alcohol. (**A**) Expression ABCB11, ABCC6, and HMGCR; (**B**) levels of cellular bile acids; (**C**) levels of conjugated bilirubin in the culture medium; (**D**) cellular lipid accumulation (red area) revealed by Oil Red O staining (400×); (**E**) quantitation of cell death; Ct, dimethyl sulfoxide as vehicle control; ERL, ritonavir, lopinavir, and alcohol; ERLG, glycyrrhizic acid and ERL; ERLU, ursodeoxycholic acid (UDCA) and ERL; *, *p* < 0.05; **, *p* < 0.01; ***, *p* < 0.005 compared to Ct.

There are several anti-cholestasis compounds/drugs being reported to protect against hepatic secretion impairments [21,22,23,24]. Glycyrrhizic acid (glycyrrhizin or GA) and ursodeoxycholic acid (UDCA) were selected to test whether they were effective against RTV, LPV, and/or alcohol-induced downregulation of ABC transporters and secretion impairments of bile acids and bilirubin in the senescent human hepatocytes. GA treatment moderately recovered the mRNA expression of ABCB11 or ABCC6 that was reduced by RTV, LPV, and alcohol (Figure 7A). The expression of HMGCR that was upregulated by RTV, LPV, and alcohol was decreased by 7% by GA treatment. In contrast, UDCA treatment increased the expression of the transporters ABCB11 and ABCC6 significantly and decreased the expression of HMGCR by 22% compared to those of senescent PHHs treated with RTV, LPV, and alcohol. The cellular accumulation of bile acids which resulted from RTV, LPV, and alcohol treatment was reduced by 4.5% by GA and reduced by 13% by UDCA (Figure 7B). The levels of direct/conjugated bilirubin in the cell culture medium were increased by 8% by GA and by 28% by UDCA, indicating a partial recovery of conjugated bilirubin secretion by either GA or UDCA (Figure 7C). The intracellular bile acid accumulation was associated with lipid accumulation inside the hepatocytes, which was observed in PHHs treated with RTV, LPV, and alcohol without GA or UDCA (Figure 7D). In the presence of glycyrrhizin, lipid accumulation indicated by Oil Red O staining was moderately reduced whereas in the presence of UDCA, the lipid accumulation was reduced much more. In addition, the anti-HIV drugs and alcohol induced about 20% cell death in senescent PHHs, which was reduced by 15% by GA treatment and by 25.8% by UCDA treatment (Figure 7E), indicating that the overall protective effects of ursodeoxycholic acid were better than the protective effects of glycyrrhizin.

## 3. Discussion

The hepatic secretion of bile acids and bilirubin is essential for the homeostasis of bile acids or bilirubin and the maintenance of digestive health in the body which is often associated with many chronic liver dysfunctions such as the abnormal accumulation of bile acids by alcohol and/or drugs. Toxic levels of bile acids may cause inflammation and cell death leading to cholestatic liver diseases [3]. It is not clear whether impaired hepatic secretion of bile acids and bilirubin by alcohol and drug exposures could deteriorate in the aging liver considering that elderly patients experience excessive use of prescription medications and a combined use of drug and alcohol. In this study, we investigated for the first time the hepatotoxic effects of antiviral drugs and/or alcohol on hepatocyte transporters and the secretion of bile acids and bilirubin in the hydrogen peroxide-induced senescent human liver cancer cell line HepG2 and doxorubicin-induced senescent primary human hepatocytes and made a few interesting preliminary findings. First, the impaired secretion of bile acids or bilirubin occurred in both HepG2 and primary human hepatocytes upon senescence or exposure to drugs and alcohol but to different degrees. In the human liver cancer cells, intracellular conjugated bilirubin increased upon senescence and extracellular conjugated bilirubin in culture medium was decreased by the antiviral drugs ritonavir and lopinavir. In the primary human hepatocytes, the status of senescence did not have a significant impact on intracellular bile acids or medium bilirubin. However, in the presence of alcohol and the two antiviral drugs, intracellular bile acids were accumulated, and medium conjugated bilirubin was decreased in senescent primary hepatocytes. These observations suggest that, in the context of hepatic secretion, cancerous hepatocytes were more sensitive to the status of senescence than to alcohol and drugs whereas normal primary hepatocytes were more sensitive to the drug and alcohol challenges than to the status of senescence. While there are reports that bile acid-induced senescence in hepatic stellate cells could modulate the behavior of liver cancer [25,26], it is not clear from this study whether the differences in bile acid profiles between HepG2 and PHHs would alter the impacts of senescence on the drug- and alcohol-induced impairments of bile acid/bilirubin secretion. It is also worth noting that although both doxorubicin and hydrogen peroxide are well known pro-oxidants that promote oxidative stress and induce cell senescence, doxorubicin at non-toxic lower doses has increasingly been used to investigate senescence/aging effects in both primary human cells and animal models that are physiologically relevant [15,16,17,27,28]. The observed senescent effects on the ABC transporters and bile acid secretion in the doxorubicin-treated primary human hepatocytes likely reflect the effects of physiological aging in human hepatocytes on hepatic secretion in vivo.

Second, there are many molecular mechanisms that could lead to the impaired hepatic secretion of bile acids and bilirubin in the liver [3,4,5,29]. In this in vitro investigation, we examined bile acid/bilirubin transporters and a couple of key enzymes and factors for bile acid synthesis, bilirubin conjugation, and drug/alcohol metabolism. We found that the intracellular accumulation of bile acids likely did not result from increased bile acid uptake since significant changes were not detected upon senescence in either HepG2 or PHHs for the relevant transporters such as the organic anion transporter polypeptide members (OATP1B1 or OATP1B3), the apical sodium-dependent bile acid transporter ASBT, and the basolateral sodium/bile acid co-transporter NTCP, which are involved in bile acid uptakes. Similarly, the reduced bilirubin secretion was not due to the reduced bilirubin conjugation required for the hepatic secretion given that the UDP-glucuronosyltransferase UGT1A1 that catalyzes the conjugation was increased by the antiviral drugs, which was likely due to the structural modification of UGT1A1 by the anti-HIV protease inhibitor ritonavir, resembling the molecular interactions between ritonavir and the human ortholog of STE24 protease observed before [30,31]. Thus, the possible causes for the intracellular bile acid accumulation might be a rise in bile acid synthesis and/or inhibition of the transporters for bile acid/bilirubin secretion. Two pieces of evidence obtained from this study support the possibilities. One is that the expression of CYP7A1 required for bile acid synthesis was increased in the control non-senescent PHHs treated with alcohol combined with the antiviral drugs, which were further increased in senescent PHHs. The other one is that expressions of adenosine triphosphate (ATP)-binding cassette (ABC) transporters (ABCB4, ABCC6, ABCB11, and ABCD3) were reduced by ritonavir and lopinavir in senescent HepG2, and the expressions of ABCB11, ABCC1, ABCC2, ABCC3, ABCC4, and ABCC6 were reduced in senescent PHHs, the latter of which were further decreased by alcohol combined with ritonavir and lopinavir. In addition, the inhibition of ABC transporters may be directly related to the senescence of hepatocytes considering that no consistent changes for other factors including CYP3A4, CYP2D6, and FXR that regulate either drug metabolism or bile acid homeostasis were detected between senescent and non-senescent hepatocytes treated with drugs and/or alcohol. It is worth investigating further the specific molecular mechanisms by which the oxidative stress-induced senescence of hepatocytes suppresses the ABC transporters for bile acid/bilirubin secretion.

Third, the transporter ABCC6 was originally known to prevent the ectopic mineralization seen in pseudoxanthoma elasticum by inducing cellular nucleotide release and pyrophosphate (PPi) [10,32]. Recent evidence suggests that ABCC6 plays an important role in maintaining cholesterol homeostasis that may indirectly affect bile metabolism and the hepatic secretion of bile acids/bilirubin [11]. Because it has been reported that knockdown of ABCC6 in HepG2 induced senescent-like cell phenotypes [20], we tested further the influence of senescence on bile acid/bilirubin secretion through knocking down ABCC6 in the primary human hepatocytes. We observed altered expressions of two senescence markers, Lamin A/C and cyclin-dependent kinase inhibitor CKI (p21), in the primary hepatocytes with ABCC6 being knocked down. Our results support the notion that ABCC6 deficiency may contribute to senescence of hepatocytes via interfering with the bile acid/bilirubin secretion. More interestingly, we also observed that expressions of CYP7A1 and hydroxy methyl glutaryl-CoA reductase (HMGCR) were increased in the PHHs with ABCC6 knockdown, which were accompanied with the accumulation of intracellular bile acids and reduced bilirubin secretion, suggesting that ABCC6 could be a cause for the severe persistent hepatocellular secretory failure observed recently in elderly patients who had ABCC6 gene mutation [9]. Thus, targeting the ABC transporters involving bile acid/bilirubin secretion might be a plausible strategy to tackle drug-/alcohol-induced hepatic secretion failure and complications. Since oxidative stress predominantly occurs in the hepatocytes due to the competition between alcohol and the drugs for the common metabolizing enzyme CYP3A4 [4] and oxidative stress promotes cell senescence and injury, we tested two potential anti-cholestasis compounds, ursodeoxycholic acid and glycyrrhizin, and showed that both ursodeoxycholic acid and glycyrrhizin partially ameliorated the impaired secretions of bile acids and bilirubin, intracellular lipid accumulation, and cell death caused by ritonavir, lopinavir, and alcohol in the primary hepatocytes deficient in ABCC6. Ursodeoxycholic acid appeared better, which might be due to its function against oxidative endoplasmic reticulum (ER) stress often associated with alcohol and drug-induced hepatotoxicity [3,4].

## 4. Materials and Methods

### 4.1. Cell Cultures, Reagents, and Treatments

HepG2 cells (HB-8065) were initially purchased from ATCC (Manassas, VA, USA) and maintained in Dulbecco’s Modified Eagle Medium (DMEM) (Cat. No.11965-092, Gibco, ThermoFisher, Walthan, MA, USA) supplemented with 10% of fetal bovine serum (FBS) (Cat. No. A3160401, Gibco, Brooklyn, NY, USA) and 1% of penicillin-streptomycin (Cat. No. 15140-122, Gibco) at a 37 °C incubator with 5% CO_2_. The medium was replaced every 3 days if not otherwise stated. Induction of senescent HepG2 was described previously [33]. Briefly, HepG2 cells were seeded in a 10 cm culture dish at a density of 4 × 10^5^/mL overnight for attachment and were then treated with 300 μM hydrogen peroxide (H_2_O_2_) for 3 consecutive days with the medium refreshed every day. When 50% to 60% of HepG2 reached senescence, the cells were allowed to recover for 3 days before re-plating them to 6-well plates at a density of 0.2 × 10^5^/well for antiviral drug treatments.

Primary human hepatocytes (PHHs) were purchased from Sigma-Aldrich (Cat. No. MTOXH1000; St. Louis, MO, USA) and maintained in a complete DMEM (ThermoFisher) containing penicillin-streptomycin (0.5%, *v*/*v*), dexamethasone (10 ppm), Gibco™ Insulin-Transferrin-Selenium (ITS-G) (1%, *v*/*v*), L-glutamine (1%, *v*/*v*), 10% of fetal bovine serum (FBS), and HEPES (15 mM). Long-term original functions of PHHs were maximally maintained with the 5C long-term culturing supplemented with forskolin, DAPT, SB431542, IWP2, and LDN193189, which was described previously [34,35]. Senescent PHHs were induced by treating the cells with 0.2 μM of doxorubicin (Cat No. D1515; Sigma-Aldrich, St Louis, MO, USA) for four days in the complete DMEM, which was refreshed every day [36]. When 50% of PHHs reached senescence, the cells were then recovered for 3 days before alcohol and/or antiviral drug treatments. The status of senescence of HepG2 and PHH was confirmed with senescence markers including expression of cyclin-dependent kinase inhibitor CKI (p21) and in situ staining of senescence-associated β-galactosidase (SA-β-gal), which were reported before [33].

Control or the senescent HepG2 were treated with antiviral protease inhibitors, ritonavir (25 μg/mL) (Cat. No. 1604803, Sigma, Burlington, MA, USA) and lopinavir (25 μg/mL) (Cat. No. 205036, Sigma), in the DMEM free of FBS for 24 h. The senescent primary liver cells were treated with the anti-HIV drugs alone at 25 μg/mL or in combination with ethanol (50 mM) in the DMEM free of FBS for 24 h. Dimethyl sulfoxide (DMSO) solution (<0.1%, *v*/*v*) was used as vehicle control for both types of hepatocytes. In some experiments, the liver cells were pre-treated with glycyrrhizic acid (Cat No. PHR1516, Sigma) at 25 µM or with ursodeoxycholic acid (Cat No. U5127; Sigma) at 100 μM in the complete DMEN medium for 24 h before being exposed to the anti-HIV drugs and/or ethanol. For bile acid or bilirubin secretion tests, the liver cells were treated with cholic acid at 1 μM (C1129, Sigma) or bilirubin at 17 μM (B4126, Sigma) in fresh DMEM free of FBS for 5 h post the anti-HIV drug and/or alcohol treatments.

To knock down expression of ABCC6 in PHHs, siRNAs of ABCC6 were applied, which were generated from the next-generation RNAi chemistry by ThermoFisher Scientific (Waltham, MA USA, Cat No. AM16708 for ABCC6 and Cat No. 4390844 for negative control). The assay ID for ABCC6 siRNA is HS01077866. Briefly, the siRNA was diluted at 30 pmol/200 μL OptiMEM and mixed with 3 μL of Lipofectamine^®^ 3000 (ThermoFisher Scientific), at 25 °C for 15 min, to which 10^5^ of PHH cells in 1 mL of antibiotics-free complete medium was added. The transfected PHHs were incubated for 48 h and then treated with ethanol or the anti-HIV drugs as described above [34].

The Lipid Oil Red O Staining Kit from Sigma (Cat No. MAK194) was applied for in situ Oil Red O staining of PHHs following the manufacturer’s instructions. Briefly, the liver cells were washed three times with cold PBS (phosphate-buffered saline) and fixed with 4% paraformaldehyde for 30 min. After the fixation, the cells were washed three times and stained with Oil Red O solution for 15 min at room temperature. Total intracellular cholesterol was measured using the Cholesterol Assay Kit from Abcam (Cat No. AB65359). To evaluate cell death, PHHs were stained with Sytox green (1 mM; Molecular Probes, Eugene, OR, USA) and counted according to previously described methods [37].

### 4.2. Measures of Bile Acids and Conjugated/Unconjugated Bilirubin

The liver cells were separated from the culture medium, washed with cold PBS, detached with 0.25% trypsin-EDTA solution from Gibco (Cat. No. 2318299), harvested at 5000 rpm for 5 min, and washed with 1 mL of 150 mM ammonium acetate (A1542-500G, Sigma) three times. The cell pellets were analyzed immediately or kept in −80 °C freezer until use. The cell media were centrifuged at 3000 rpm for 20 min at 4 °C to remove debris, and the supernatant was collected for analysis or kept in −80 °C freezer.

The total bile acid assay kit from BioSource (Cat No. MBS169245, Los Angeles, CA, USA) was used to measure total bile acids within PHH cells, and the total bilirubin ELISA kit (MBS166923) was used to measure total or direct (conjugated) bilirubin in the PHH culture media according to the manufacturer’s instructions.

The Liquid Chromatography–Mass Spectrum (LC–MS) at USC Mann Multi-Omics Mass Spectrometry Core in Los Angeles was used to measure unconjugated bilirubin (UCB) and conjugated bilirubin monoglucuronide (BMG) in HepG2 cells or culture media [38,39]. The cells (20–50 mg) were suspended in 50 µL of MS grade H_2_O (Cat No. W6-4, Fisher Scientific) containing ascorbic acid at 36.6 mM (Cat No. A0278-100G, Sigma), centrifuged at 1350 rpm for 5 min, and resuspended in 200 µL of cold MS grade MeOH (Cat No. A452-4, Fisher Scientific). The cells were then vortexed at maximum speed for 10 s and shaken at 2000 rpm for 5 min for complete lysis on a shaker covered with aluminum foil. The lysates were briefly centrifuged and mixed with 50 µL of DMSO (Cat No. 85190, Thermo Scientific, Waltham, MA USA) containing mesobilirubin at 5.8 µM (as internal standard, IS) (Cat No. sc-263467, Santa Cruz, CA, USA), which were then vortexed for 10 s, shaken at 2000 rpm for 5 min, sonicated for 10 min in an ice bath, and finally incubated at −20 °C for 15 min for protein precipitation. After centrifugation at 18,000× *g* for 5 min at room temperature, 100 µL of supernatant was collected to an amber vial (Cat No. 60180-1655, Thermo Scientific) for MS analysis. The culture medium sample processing was similar to the cell samples except that 15 mL of medium was mixed with 35 μL of MS grade H_2_O containing ascorbic acid at 52.3 mM.

A measurement of 10 µL of each sample or bilirubin standard (from 1 µM to 1 nM) was injected onto a Luna Omega PS C18, 2.1 × 150 mm 1.6 µm column (00F-4752-AN; Phenomenex, Torrance, CA, USA) and eluted using a 1290 Infinity II HPLC system (Agilent, Santa Clara, CA, USA). The mobile phases were A: ammonium formate at 5 mM adjusted to pH 3, and B: 2.5:7.5:90 of ammonium formate at 200 mM (Cat No. 014517.18, Fisher Scientific) adjusted to pH 3: H_2_O: acetonitrile. The flow rate was 0.5 mL/min and applied to the following gradient–time (min)/%B: 0/42, 3.5/42, 6.5/97, 10.6/97, 10.7/42, and 14/42. The autosampler and column compartment were maintained at 6 °C and 35 °C, respectively.

The effluent from the column was directed to an IonDrive Turbo V source (Ab Sciex, Framingham, MA, USA) leading to a 6500+ Qtrap mass spectrometer (AB Sciex) acquiring mass spectra in the positive polarity in scheduled MRM mode. The source was set at the following settings: CUR gas at 30 psi, CAD set to medium, IS 5500V, heater at 400 °C, and GS1 and GS2 both 40 psi. The positional analog impurities from the bilirubin standard being detected were less than 0.5% (Supplementary Figure S2). Table 1 shows the compound parameters and retention times.

### 4.3. Real Time RT-PCR, Immunoblot, Microarray, and Enzyme Activity Assays

Portions of the liver cells detached with the 0.05% Trypsin-EDTA solution were used for RNA isolation or protein extraction. Total RNA was extracted using the RNeasy Mini Kit from Qiagen (Cat No. 74136, Venlo, The Netherlands) and reverse transcription was performed using QuantiTect Reverse Transcription Kit from Qiagen (Cat No. 205311) following the manufacturer’s instructions. The KiCqStart One Step Probe RT-qPCR Readymix or Ready Mix Taq PCR Kit from Sigma (Cat No. KCQS07) was used for semiquantitative PCR. The PCR primers are listed in Supplementary Materials (Table S1), and the PCR products were quantified with the Delta Ct methods described before [33,37]. Extraction of proteins and immunoblotting analysis were conducted according to the method described before [33,34]. The antibodies and company catalog numbers are provided in Supplementary Materials (Table S2). Quantitative immunoblot analysis was conducted with ImageJ (NIH, USA; https://imagej.net/ij/; accessed on 2 March 2025) using GAPDH or a-tubulin as normalization control.

Total RNA for in situ hybridization and microarray assays was isolated from PHH cells using the Qiagen RNeasy Mini Kit and 500U of RNase inhibitor from Millipore-Sigma (Cat No. R7397) following the manufacturer’s instructions. Microarray data were processed and analyzed with the Illumina Bead Studio software 3.2 described previously [34,37]. Data of the average signal were filtered with a *p*-value (<0.05) and normalized via rank invariant normalization, after which gene expression changes were clustered for liver cell transporters and bile acid/bilirubin metabolism and exported for heat-mapping comparisons.

Membrane proteins were extracted using the Membrane Protein Extraction Kit from ThermoFisher Scientific (Cat No. 89842) and enzyme activity of UDP-glucuronosyltransferase (UGT) was analyzed using the UGT Activity Assay Kit from Abcam (Cat No. ab273331) following the manufacturer’s instructions.

### 4.4. Statistical Analysis

The experiments were performed in triplicates. Data are presented as means ± SD unless otherwise indicated. Statistical analyses were performed with GraphPad Prism^®^ 6 using the *t*-test for comparison of two groups and the ANOVA for comparing multiple groups. The level of significance was set at *p* < 0.05.

## 5. Conclusions

The hepatocyte excretion of bile acids and bilirubin is an important physiological process that is critical for preventing the toxic accumulation of bile acids and bilirubin and subsequent injury in the liver and other organs. We demonstrated that senescence of hepatocytes in vitro due to oxidative stress had adverse effects on ABC transporters including ABCB11, ABCC1, ABCC2, ABCC3, ABCC4, and ABCC6 that function in the hepatic secretion of bile acids or bilirubin, which become much worse in the presence of antiviral drugs combined with alcohol but could be improved by the anti-cholestasis agent ursodeoxycholic acid which functions against oxidative ER stress. Also, targeting the ABC transporters involving bile acid/bilirubin secretion might be a plausible strategy to tackle drug-/alcohol-induced hepatic secretion failure and its complications.

## Figures and Tables

**Table 1:** Compound parameters and retention times.

Compound	Retention Time (min)	Precursor *m*/*z*	Product *m*/*z*	DP (V)	EP (V)	CE (eV)	CXP (V)
Bilirubin (+)	8.92	585	299	86	10	28.5	10
BMG (+)	5.6	761.3	475.3	100	10	26	10
Mesobilirubin_IS (+)	9.31	589.4	301.3	80	10	26	10

MS data was acquired using Analyst software version 1.7 (AB Sciex LLC., Redwood, CA, USA) and additionally peaks were integrated and QC’d using Analyst.

## Data Availability

The original contributions presented in this study are included in the article/Supplementary Material. Further inquiries can be directed to the corresponding author.

## Supplementary Materials

The following supporting information can be downloaded at https://www.mdpi.com/article/10.3390/ijms27031189/s1.

## Abbreviations

The following abbreviations are used in this manuscript:

ABC Transporters	adenosine triphosphate (ATP)-binding cassette transporters
BMG	conjugated bilirubin monoglucuronide
CYP7A1	the cytochrome P450 enzyme 7A1
ER	endoplasmic reticulum
FXR	nuclear farnesoid X receptor
GA	glycyrrhizic acid or glycyrrhizin
HMGCR	hydroxy methyl glutaryl-CoA reductase
LC–MS	Liquid Chromatography–Mass Spectrum
LPV	lopinavir
MASLD	metabolic dysfunction-associated steatotic liver disease
OATP1B1 and OATP1B3	the organic anion transporting polypeptide 1B1 and 1B3
P21	cyclin-dependent kinase inhibitor CKI
PHHs	primary human hepatocytes
PHSF	severe persistent hepatocellular secretory failure
PXE	pseudoxanthoma elasticum
RTV	ritonavir
SA-β-gal	senescence associated b-galactosidase
UCB	cellular un-conjugated bilirubin
UDCA	ursodeoxycholic acid
UGT1A1	the enzyme glucuronyltransferase 1A1
