# Supplementary material for: Effects of Ritonavir, Lopinavir, and Alcohol on ABC Transporters and Secretion of Bile Acid and Bilirubin in Senescent Hepatocytes
Source: Int J Mol Sci. 2026 Jan 25;27(3):1189. doi: 10.3390/ijms27031189 (PMC12897732; doi:10.3390/ijms27031189)
Supplement: Supplementary file 1 [file ijms-27-01189-s001.zip › ijms-4069994-supplementary.pdf]

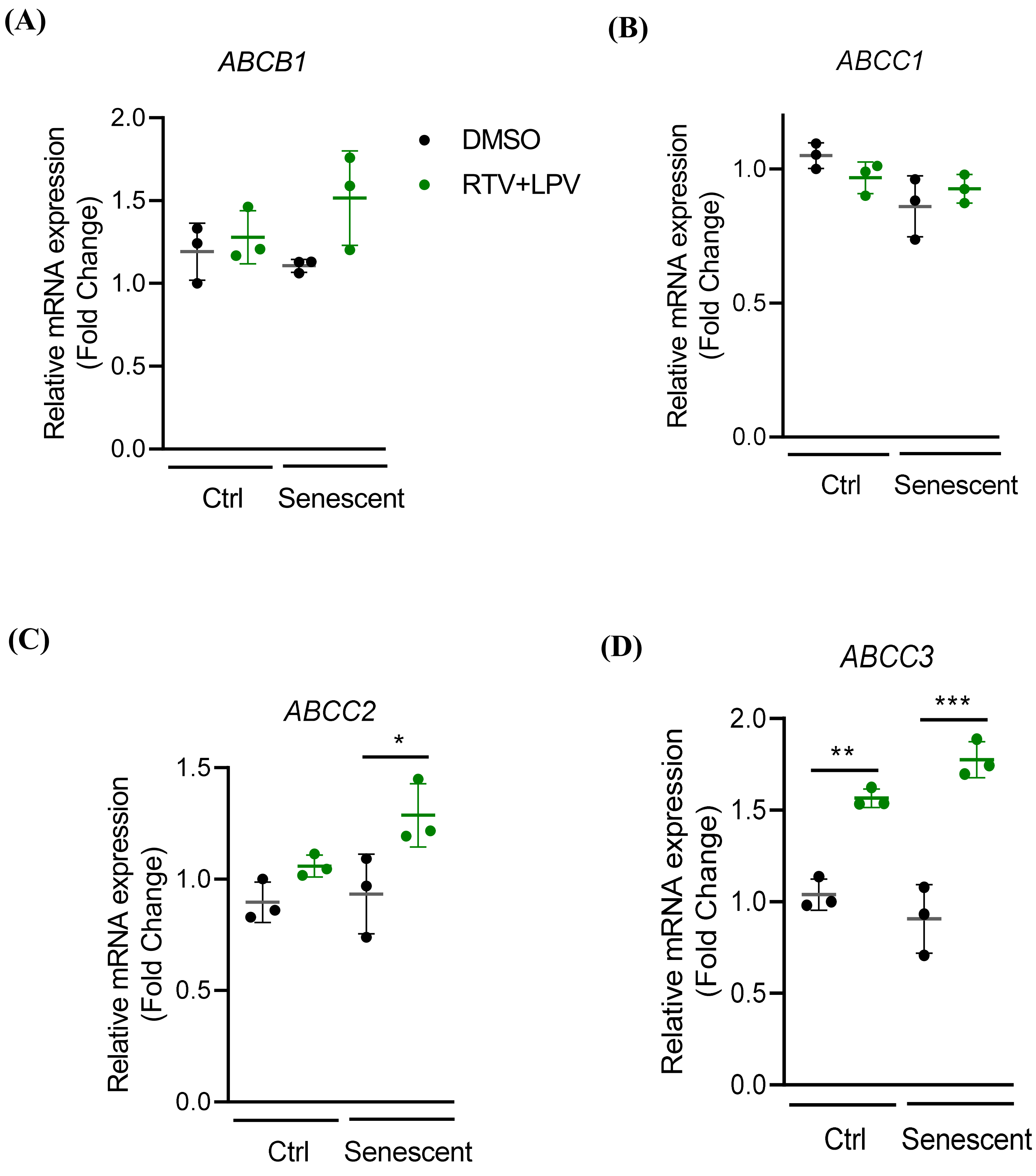

Figure S1 Effects of ritonavir (RTV) and lopinavir (LPV) on expression of ABC transporters in senescent HepG2.

Ctrl, non-senescent control cells; DMSO, dimethyl sulfoxide as vehicle control. *, $p<0.05$; **, $p<0.01$; ***, $p< 0.005$.

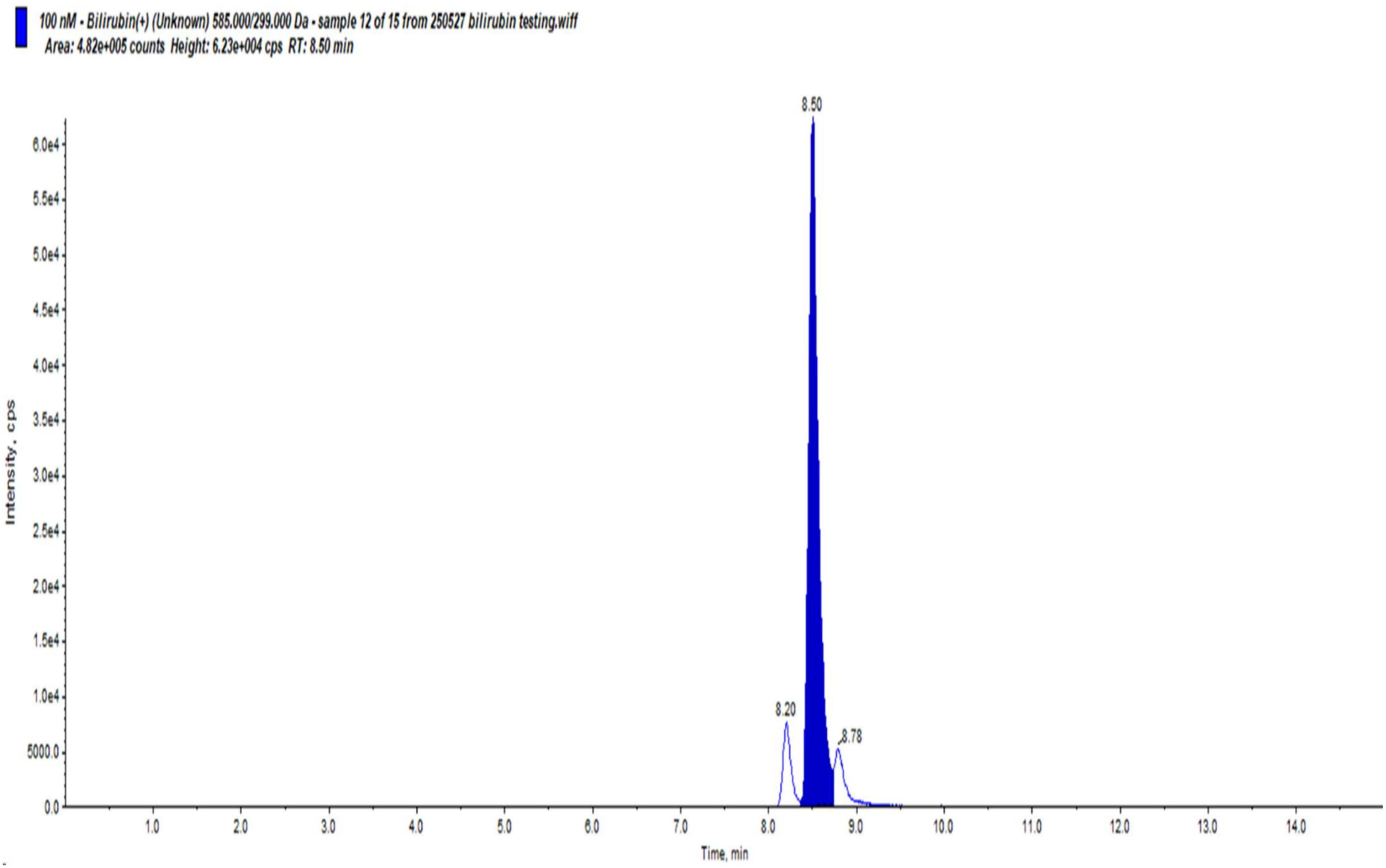

Figure S2 Detection of bilirubin on a 5500-mass spectrometer.
The ratio of the peak areas of Bilirubin (BR) to Biliverdin (BV) was about 200 to 1, indicative of very little conversion of BR to BV.

Table S1 Sources and catalog numbers for antibodies

Name	Sources	Catalog No.
β-actin	Millipore-Sigma	A5441
ABCB4	ThermoFisher	MA5-37748
ABCB11	Novusbio	NBP3-27037
ABCC3	Abcam	ab226804
ABCC6	CellSignaling	10666
ABCG5	ThermoFisher	PA5-97127
GAPDH	Proteintech	60004-1-Ig
HMGCR	Sigma	AMAB90618
FXR	ThermoFisher	BS-12867R
CYP7A1	ThermoFisher	PA5-15216
Lamin A/C	Abcam	ab224816
OATP1B1	Abcam	ab254575
OATP1B3	Proteintech	66381-1-Ig
p21	Abcam	ab109199
α-Tubulin	Sigma	T6199-100ul
UGT1A1	Proteintech	23495-1-AP

Table S2 PCR Primer Pairs for all genes tested

Gene Name	Primer Sequences (5'---3')		Gene Bank
	Forward	Reverse	Access no.
ABCB1	GCTGTCAAGGAAGCCAATGCCT	TGCAATGGCGATCCTCTGCTTC	NM_000927
ABCB4	ATCCTCACCAGAAGACTGCGGT	GCAGCATCTGTGGCAAGTCTTG	NM_000443
ABCB11	AGCCACACAGACCAGGATGTTG	CAATGAACCGCCTCTCCTTTCC	NM_003742
ABCC1	CCGTGTACTCCAACGCTGACAT	ATGCTGTGCGTGACCAAGATCC	NM_004996
ABCC2	GCCAACTTGTGGCTGTGATAGG	ATCCAGGACTGCTGTGGGACAT	NM_000392
ABCC3	GAGGAGAAAGCAGCCATTGGCA	TCCAATGGCAGCCGCACTTTGA	NM_003786
ABCC4	CTGTTGGAGGATGGTGATCTGAC	CTGCTAACTTCCGCATCTACTGC	NM_005845
ABCC6	GCCCAGAGACTTAGCGACAG	GGCGAATCCAAGCACCATCT	NM_001171
ABCD3	GTTCCTTTAGCAACGCCAAATGG	CTCTTTCCGCAGCCATTTGGAC	NM_002858
ABCG5	GATTGTCGTCCTCCTGGTGGAA	TCTCCGAAGCTCAGGATGGCAA	NM_022436
CYP3A4	CCGAGTGGATTTCCTTCAGCTG	TGCTCGTGGTTTCATAGCCAGC	NM_017460
CYP7A1	CAAGCAAACACCATTCCAGCGAC	ATAGGATTGCCTTCCAAGCTGAC	NM_000780
CYP2D6	GCAAGAAGTCGCTGGAGCAGTG	CTCACGGCTTTGTCCAAGAGAC	NM_000106
GAPDH	CATGGCCTTCCGTGTTCCT	GCGGCACGTCAGATCCA	NM_0012567 99
p21	CCTGGTGATGTCCGACCTG	CCATGAGCGCATCGCAATC	BC000312
OATP1B1	TTGCACTGGGTTTCCACTCA	AAGCCCAAGTAGACCCTTGAAA	NM_006446
OATP1B3	GTCACCTTGTCTAGCAGGATGC	GCATTCACCCAAGTGTGCTGAG	NM_019844
18S rRNA	CAGCCACCCGAGATTGAGCA	TAGTAGCGACGGGCGGTGTG	NR_145820.1
UGT1a1	GCAAAGCGCATGGAGACTAAGG	GGTCCTTGTGAAGGCTGGAGAG	NM_000463
